# Preoperative differentiation of gastric schwannomas and gastrointestinal stromal tumors based on computed tomography: a retrospective multicenter observational study

**DOI:** 10.3389/fonc.2024.1344150

**Published:** 2024-03-05

**Authors:** Luping Zhao, Guanjie Cao, Zhitao Shi, Jingjing Xu, Hao Yu, Zecan Weng, Sen Mao, Yueqin Chen

**Affiliations:** ^1^Department of Medical Imaging, The Affiliated Hospital of Jining Medical University, Jining, Shandong, China; ^2^Department of Radiology, Guangdong Provincial People’s Hospital, Guangdong Academy of Medical Sciences, Guangzhou, China; ^3^Department of Ultrasound, The Affiliated Hospital of Jining Medical University, Jining, Shandong, China

**Keywords:** gastric schwannoma, gastrointestinal stromal tumor, computed tomography, diagnosis, nomogram

## Abstract

**Introduction:**

Gastric schwannoma is a rare benign tumor accounting for only 1–2% of alimentary tract mesenchymal tumors. Owing to their low incidence rate, most cases are misdiagnosed as gastrointestinal stromal tumors (GISTs), especially tumors with a diameter of less than 5 cm. Therefore, this study aimed to develop and validate a diagnostic nomogram based on computed tomography (CT) imaging features for the preoperative prediction of gastric schwannomas and GISTs (diameters = 2–5 cm).

**Methods:**

Gastric schwannomas in 47 patients and GISTs in 230 patients were confirmed by surgical pathology. Thirty-four patients with gastric schwannomas and 167 with GISTs admitted between June 2009 and August 2022 at Hospital 1 were retrospectively analyzed as the test and training sets, respectively. Seventy-six patients (13 with gastric schwannomas and 63 with GISTs) were included in the external validation set (June 2017 to September 2022 at Hospital 2). The independent factors for differentiating gastric schwannomas from GISTs were obtained by multivariate logistic regression analysis, and a corresponding nomogram model was established. The accuracy of the nomogram was evaluated using receiver operating characteristic and calibration curves.

**Results:**

Logistic regression analysis showed that the growth pattern (odds ratio [OR] 3.626; 95% confidence interval [CI] 1.105–11.900), absence of necrosis (OR 4.752; 95% CI 1.464–15.424), presence of tumor-associated lymph nodes (OR 23.978; 95% CI 6.499–88.466), the difference between CT values during the portal and arterial phases (OR 1.117; 95% CI 1.042–1.198), and the difference between CT values during the delayed and portal phases (OR 1.159; 95% CI 1.080–1.245) were independent factors in differentiating gastric schwannoma from GIST. The resulting individualized prediction nomogram showed good discrimination in the training (area under the curve [AUC], 0.937; 95% CI, 0.900–0.973) and validation (AUC, 0.921; 95% CI, 0.830–1.000) datasets. The calibration curve showed that the probability of gastric schwannomas predicted using the nomogram agreed well with the actual value.

**Conclusion:**

The proposed nomogram model based on CT imaging features can be used to differentiate gastric schwannoma from GIST before surgery.

## 1 Introduction

Following the broad applicability of esophagogastroduodenoscopy and endoscopic ultrasonography, the detection rate of gastric tumors smaller than 5 cm in diameter increased (1). Therefore, it is important to diagnose accurately and develop therapeutic strategies for small gastric lesions. The major categories of gastric submucosal tumors (SMTs) are stromal, neurogenic, and myogenic tumors (2). Different pathological types of SMTs exhibit different biological behaviors (3). Gastric schwannoma is a rare, slow-growing, benign tumor that mostly arises from Schwann cells in the nerve sheaths of the intermuscular nerve plexus of the stomach and accounts for only 1–2% of alimentary tract mesenchymal tumors. Owing to their low incidence rate, the clinical misdiagnosis rate is as high as 96.7% (4), and most cases are misdiagnosed as gastrointestinal stromal tumors (GISTs). The common computed tomography (CT) imaging features of high-risk GISTs include size ≥5 cm, extraluminal or mixed growth pattern, lobulated contour, heterogeneous enhancement, hypo-enhancement, necrosis, and enlarged feeding vessels (5, 6), making it easier to distinguish from gastric schwannoma. However, for low-risk GIST or atypical imaging features with intermediate- to high-risk GIST with diameters less than 5 cm (7), they usually have CT features similar to gastric schwannoma, and gastric schwannoma is almost always diagnosed as GIST before surgery. Although there are no significant differences or specificities in their clinical characteristics, their treatment methods and prognosis differ (8). Owing to the low malignant potential of gastric schwannoma, endoscopic resection is an effective and safe treatment method, with excellent follow-up results and prognosis (9). However, 10–30% of GISTs are considered potentially malignant tumors exhibiting recurrent and metastatic characteristics. Complete surgical resection is an effective method, but the selection of surgical methods needs to be comprehensively considered. Therefore, the accurate distinction of gastric schwannoma from GIST before surgery is crucial not only in the selection of a clinical plan but also in treatment and prognosis.

Although endoscopic ultrasound examination and endoscopic ultrasound-guided tissue sampling have become important tools for distinguishing solid tumors, including gastrointestinal tumors (10, 11), they are invasive and depend on the skills of the operator and may have limitations in evaluating extraluminal growth tumors, lymph nodes, and the relationship between tumors and adjacent structures. CT is a non-invasive and economical imaging method that can clarify the location, size, growth pattern, adjacent organs, blood supply, and distant metastasis of tumors (12). Recently, most studies have differentiated gastric schwannoma from GIST based on qualitative or quantitative descriptions of CT imaging features or the construction of scoring systems (13–15), but the results vary. A recent study (16) showed that the model based on CT qualitative and quantitative features helps distinguish between gastric schwannoma and GIST using machine learning methods, but the model is difficult to apply in clinical practice. Therefore, we aimed to construct a nomogram prediction model based on CT image features to facilitate the preoperative differential diagnosis of gastric schwannoma and GIST and provide suggestions for clinical decision-making.

## 2 Materials and methods

### 2.1 Patients

**Figure 1:**
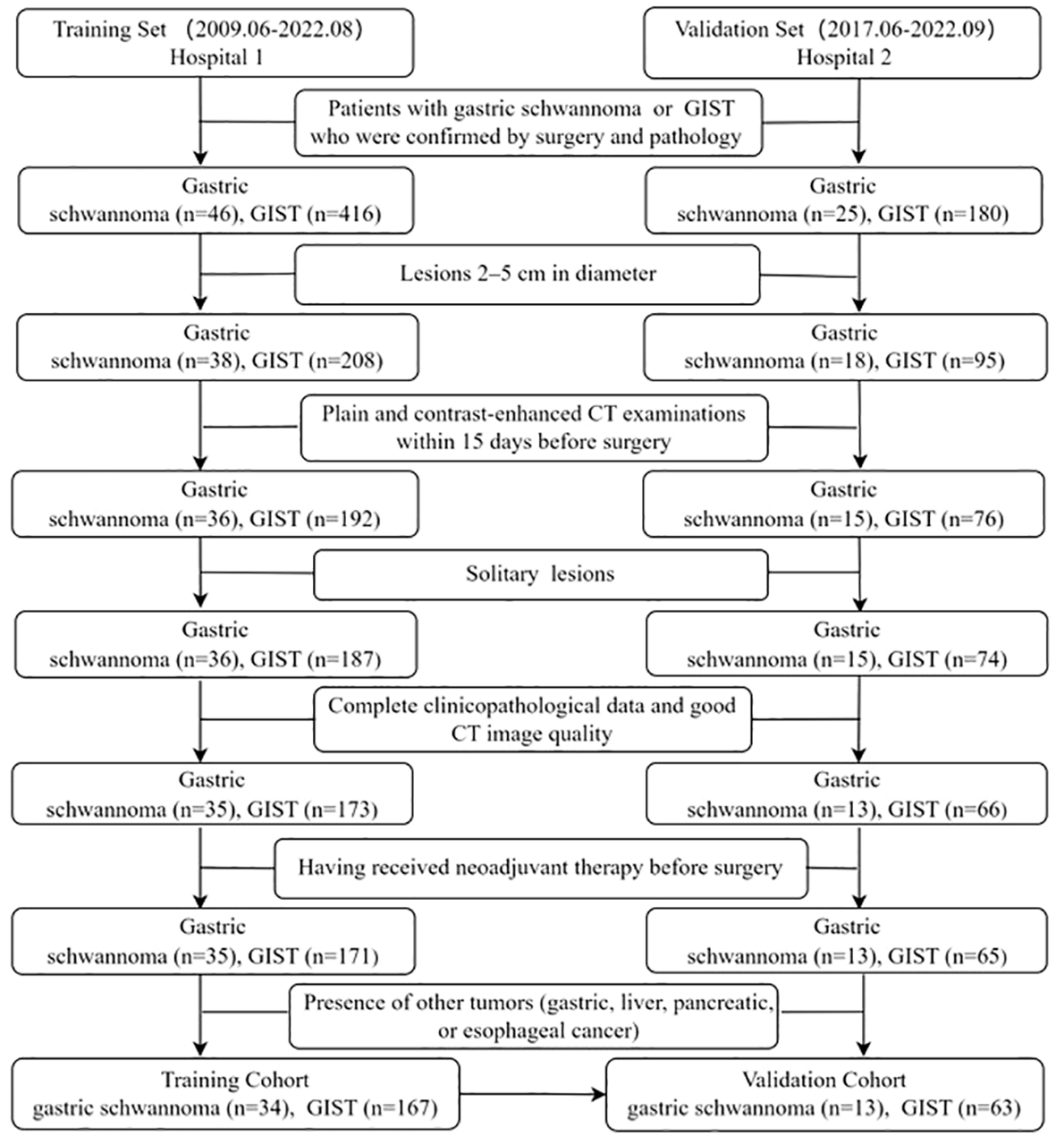
Flow chart illustrating the patient selection process.

This retrospective study was approved by the institutional Ethics Review Board of the Affiliated Hospital of Jining Medical University, and the requirement for written informed consent was waived by the Review Board. The study enrolled 277 patients with gastric schwannomas or GISTs from two independent hospitals. From June 2009 to August 2022, a total of 246 patients with gastric schwannoma and GIST (diameter=2-5cm) confirmed by postoperative histopathology and immunohistochemistry were recruited in The Affiliated Hospital of Jining Medical University (Hospital 1). Two gastric schwannoma and Sixteen GIST patients were excluded due to lack of preoperative CT data, five GIST patients were excluded due to the presence of two or more lesions, one gastric schwannoma and seven GIST patients were excluded due to poor image quality, seven GIST patients were excluded due to lack of clinical data, two GIST patients were excluded due to preoperative adjuvant therapy, one gastric schwannoma patient complicated with esophageal cancer, and four GIST patients complicated with gastric, liver, pancreatic cancers and gastric leiomyoma were excluded. Finally, 34 patients with gastric schwannomas and 167 patients with GISTs were consecutively included in the training set to determine the CT image features for differentiating gastric schwannomas from GISTs and construct a nomogram model. The inclusion criteria were as follows: (1) lesions 2–5 cm in diameter, (2) plain and contrast-enhanced CT examinations within 15 days before surgery, (3) solitary lesions, and (4) complete clinicopathological data and good CT image quality. The exclusion criteria were as follows: (1) having received neoadjuvant therapy before surgery and (2) presence of other tumors (gastric, liver, pancreatic, or esophageal cancer). An external validation set of 13 patients with gastric schwannomas and 63 with GISTs was acquired using the same criteria from June 2017 to September 2022 in Guangdong Provincial People’s Hospital (Hospital 2) to validate the performance of the nomogram model. Details of the enrolled patients are shown in **Figure 1**.

GISTs >5 cm had a higher risk of malignant behavior and were more likely to differentiate from other gastric SMTs. However, gastric schwannomas usually had CT features similar to those of GISTs and were nearly always preoperatively diagnosed as GISTs, especially tumors with diameters less than 5 cm. GISTs had potential risks of metastasis and recurrence. The National Comprehensive Cancer Network guidelines recommend that all GISTs more than 2 cm in diameter must be resected (17). However, there remains some controversy regarding the surgical methods and resection ranges of GIST with a diameter of 2–5 cm (18, 19). Therefore, this study selected tumors with a maximum diameter of 2–5 cm as the research objects.

### 2.2 CT image acquisition

CT examinations were performed on a multidetector-row CT scanner (Siemens SOMATOM Definition, Siemens Healthcare, City, Germany) in Hospital 1 and on a 256-slice CT scanner (Brilliance iCT, Philips Medical Systems, The Netherlands) and a Lightspeed VCT scanner (GE Healthcare, Chicago, IL) in Hospital 2. The CT parameters were as follows: tube voltage = 120 kV, tube current =150–230 mA, 512 × 512 matrix, tube rotation time = 0.5–0.8 s, field of view of 350 × 350 mm, pitch = 0.6, section thickness = 5 mm, and 1 mm reconstruction interval. Before the CT examination, the patients were required to fast for 6–8 h. Water (500–1000 ml) was orally administered for 5 min before the scan. Subsequently, 80–100 ml non-ionic iodinated contrast medium (350 or 370 mg I/ml) was injected through the median cubital vein using a double-barbed high-pressure syringe at flow rates of 3.0–3.5 ml/s. The arterial, portal, and delayed phases were performed at 25–30, 60–65, and 120–140 s after contrast injection, respectively.

### 2.3 Imaging analysis

All images were independently and retrospectively reviewed by two abdominal radiologists with 10 and 5 years of experience blinded to the clinical data and pathological information. Any inconsistency was resolved by consultation with senior supervisors (with 16 years of experience).

All tumors were evaluated for the following CT features: 1) tumor location: the upper, central, and lower parts of the stomach were divided by lines which connected the trisected points on the lesser and greater curvatures (20); 2) contour: regular (round or oval) or irregular; 3) growth pattern: intraluminal, extraluminal, or mixed (21); 4) necrosis, ulceration and calcification: present or absent; 5) tumor vessels: present or absent; 6) tumor-associated lymph nodes: lymph nodes which were enlarged in the fatty space around the tumor (which were confirmed by surgery and pathology) or became small or disappeared after postoperative follow-up, recorded as present or absent; 7) enhancement pattern: homogeneous or heterogeneous (22); 8) enhancement degree: quantitatively evaluated by the difference between the CT values of the enhanced (the larger of either venous phase or delayed phase) and the non-enhanced phases images on the same anatomical slice—a difference <20 Hounsfield units (HUs) was defined as mild enhancement; values of 20–40 HUs were considered as moderate enhancement and >40 HUs as strong enhancement (11); owing to the small number of mild enhancement cases, these were classified as moderate enhancement cases and collectively referred to as mild to moderate enhancement; 9) long diameter (LD)/short diameter (SD) ratio: LD and SD of the central slice of each mass were the maximum and minimum values that were independently measured on CT images in three different orientations (axial, coronal, and sagittal) and LD/SD ratio was calculated (23); and 10) circular regions of interests (ROIs with areas of 16–20 mm^2^ and avoiding areas of cystic lesions, calcifications, ulcers, or tumor vessels) were placed at three different homogeneous sites of the lesion and then averaged out, including CT values during non-enhanced (CTV-N), arterial (CTV-A), portal (CTV-P), and delayed phases (CTV-D), were recorded (18).

### 2.4 Statistical analysis

All statistical analyses were performed using the Statistical Package for Social Sciences software (IBM SPSS Statistics Version 26.0, IBM Corp, Armonk, NY, USA) and R software (version 4.1.3; http://www.R-project.org). The conformity of the variables to the normal distribution was examined using the Shapiro–Wilk test. Normally distributed continuous variables were quantified and reported as the mean ± standard deviation, non-normally distributed variables as median values (Q1, Q3), and categorical data as frequencies (percentages). For quantitative analysis, Student’s t-test was used for normally distributed continuous variables, and the Mann–Whitney U test was used for data that were not normally distributed. For qualitative analysis, the Chi-square test or Fisher’s exact test was used for categorical variables. The intra-class correlation coefficient (ICC) was calculated for the inter-observer agreement of continuous variables. Good consistency was considered when 0.75 ≤ ICC ≤ 1 (24). Each variable that was statistically significant in the univariate analysis (P<0.05) was subjected to collinearity assessment and logistic regression analysis with a forward stepwise approach to confirm independent influencing factors in differentiating gastric schwannoma and GIST, which were used to construct a nomogram. Calibration was evaluated using the Hosmer–Lemeshow goodness-of-fit test, receiver operating characteristic analysis was performed using the DeLong method, and the area under the curve (AUC) was used to evaluate the diagnostic efficiency of the model in the training and validation sets. All outcomes were considered statistically significant if P<0.05.

## 3 Results

### 3.1 Clinicopathological characteristics

The clinicopathological characteristics of patients in the training and validation sets are listed in **Table 1**. A total of 201 patients, comprising 34 with gastric schwannomas (5 men and 29 women, age 30–80 years, mean 60.38 ± 11.43 years) and 167 with GISTs (66 men and 101 women, age 33–85 years, mean 61.74 ± 11.5 years), were enrolled as the training set. The proportion of female patients in the gastric schwannoma group (29/34, 85.29%) was significantly higher than that in the GIST group (101/167, 60.48%) (P=0.006). However, there were no significant differences in age (P=0.525) or clinical symptoms (P=0.405) between the two groups.

**Table 1:** Clinical characteristics data of patients in training and validation cohort.

Clinical Characteristics	Training cohort (n=201)			Validation cohort(n=76)		
	Gastric schwannoma (n=34)	GIST (n=167)	*P value*	Gastric schwannoma (n=13)	GIST (n=63)	*P value*
**Age (years)**	60.38±11.43	61.74±11.35	0.525	53.85±14.63	62.49±10.33	0.016
**Sex**			0.006^a^			0.923^a^
**Male**	5(14.71%)	66(39.52%)		6(46.15%)	30(47.62%)	
**Female**	29(85.29%)	101(60.48%)		7(53.85%)	33(52.38%)	
**Clinical** **Symptoms**			0.405^a^			0.994^a^
**Present**	19(55.88%)	106(63.47%)		7(53.85%)	34(53.97%)	
**Absent**	15(44.12%)	61(36.53%)		6(46.15%)	29(46.03%)	
Risk category						
**Very low /Low risk**		118(70.66%)			46(73.02%)	
**Intermediate risk**		36(21.56%)			15(23.81%)	
**High risk**		13(7.78%)			2(3.17%)	

Independent samples t tests were applied in continuous variables.

a Chi-squared tests were used in categorical variables.

Seventy-six patients were included in the validation set (13 with gastric schwannomas, 6 men and 7 women, age 31–86 years, mean 53.85 ± 14.63 years; 63 with GISTs, 30 men and 33 women, age 40–79 years, mean 62.49 ± 10.33 years). The average age of patients in the gastric schwannoma group was slightly higher than that in the GIST group, and there was a significant difference between the mean ages of the two groups (P=0.016). However, there were no significant differences between men and women (P=0.923) or in clinical symptoms between groups (P=0.994). The clinical symptoms of patients in the training and validation sets were mainly abdominal pain and discomfort, and rare symptoms included melena and hematemesis. Asymptomatic patients were mostly diagnosed during physical examination.

### 3.2 Inter-observer agreement

The inter-observer agreement in the training and validation cohorts is shown in **Table 2**. The overall inter-observer agreement for measurements of all continuous variables was excellent in the training and validation sets.

**Table 2:** Inter-observer agreement in training and validation cohort.

variables	Training cohort		Validation cohort	
	ICC value	95 %CI	ICC value	95 %CI
**LD**	0.899	0.860–0.926	0.935	0.878–0.963
**SD**	0.964	0.952–0.972	0.921	0.839–0.957
**CTV-N**	0.802	0.669–0.874	0.820	0.730–0.882
**CTV-A**	0.883	0.795–0.927	0.916	0.870–0.946
**CTV-P**	0.909	0.882–0.931	0.925	0.885–0.952
**CTV-D**	0.940	0.922–0.954	0.932	0.894–0.956

LD, SD indicate the tumor of long diameter and short diameter, respectively. CTV-N, CTV-A, CTV-P, and CTV-D CT values during nonenhanced, arterial, portal, and delayed phases, respectively.

### 3.3 CT imaging features

A comparison of the CT imaging features in the training and validation sets is shown in **Table 3**. In the qualitative analysis, tumor location (P=0.003), contour (P=0.003), growth pattern (P=0.001), absence of necrosis (P=0.001), presence of tumor-associated lymph nodes (P<0.001), enhancement pattern (P=0.002), and degree of enhancement (P=0.006) were significantly different between the gastric schwannoma and GIST groups in the training set. However, there were no significant differences in tumor vessels (P=0.944), ulceration (P=0.813), or calcification (P=0.900) between the two groups in the training set. In the validation set, there were significant differences in tumor location (P=0.005), absence of necrosis (P=0.015), presence of tumor-associated lymph nodes (P <0.001), and degree of enhancement (P=0.02) between the two groups. However, there were no significant differences in contour (P=1.0), growth pattern (P=0.767), tumor vessels (P=0.379), ulceration (P=1.0), calcification (P=0.582), or enhancement pattern (P=0.692) between the two groups in the validation set.

**Table 3:** CT Imaging Features in training and validation cohort.

CT Imaging Features	Training cohort (n=201)			Validation cohort(n=76)		
	Gastric schwannoma (n=34)	GIST (n=167)	*P value*	Gastric schwannoma (n=13)	GIST (n=63)	*P value*
**Location**			0.003			0.005
**Upper Stomach**	5(14.71%)	73(43.71%)		2(15.38%)	30(47.62%)	
**Central Stomach**	21(61.76%)	77(46.11%)		7(53.85%)	30(47.62%)	
**Lower Stomach**	8(23.53%)	17(10.18%)		4(30.77%)	3(4.76%)	
**Contour**			0.003			1.0
**Regular**	29(85.29%)	97(58.08%)		10(76.92%)	46(73.02%)	
**Irregular**	5(14.71%)	70(41.92%)		3(23.08%)	17(26.98%)	
**Growth pattern**			<0.001			0.308
**Intraluminal Type**	7(20.59%)	91(54.49%)		5(38.46%)	34(53.97%)	
**Extraluminal or** **Mixed Type**	27(79.41%)	76(45.51%)		8(61.54%)	29(46.03%)	
**Necrosis**			0.001			0.015^a^
**Present**	9(26.47%)	97(58.08%)		0(0%)	21(33.33%)	
**Absent**	25(73.53%)	70(41.92%)		13(100%)	42(66.67%)	
**Calcification**			0.900			0.582^a^
**Present**	6(17.65%)	31(18.56%)		0(0%)	6(9.52%)	
**Absent**	28(82.35%)	136(81.44%)		13(100%)	57(90.48%)	
**Ulceration**			0.813			1.0
**Present**	9(26.47%)	41(24.55%)		3(23.08%)	17(26.98%)	
**Absent**	25(73.53%)	126(75.45%)		10(76.92%)	46(73.02%)	
**Tumour-Associated** **Lymph Node**			<0.001			<0.001
**Present**	18(52.94%)	8(4.79 %)		6(46.15 %)	3(4.76 %)	
**Absent**	16(47.06%)	159(95.21%)		7(53.85 %)	60(95.24 %)	
**Intratumoral** **enlarged vessels**			0.944			0.379
**Present**	11(32.35%)	53(31.74%)		6(46.15%)	21(33.33%)	
**Absent**	23(67.65%)	114(68.26%)		7(53.85%)	42(66.67%)	
**Enhancement** **Degree**			0.006			0.02
**Mild to Moderate** **enhancement**	16(47.06%)	119(71.26%)		6(46.15%)	49(77.78%)	
**Strong enhancement**	18(52.94%)	48(28.74%)		7(53.85%)	14(22.22%)	
**Enhancement pattern**			0.002			0.692
**Homogeneous**	22(64.71%)	65(38.92%)		8(61.54%)	35(55.56%)	
**Heterogeneous**	12(35.29%)	102(61.08%)		5(38.46%)	28(44.44%)	
**LD/SD Ratio**	1.27±0.21	1.32±0.23	0.276	1.33±0.28	1.32±0.19	0.600
**CTV-N**	35.32±4.31	32.92±6.28	0.034	35.15±5.23	36.02±5.79	0.814
**CTV-A**	49.62±9.17	52.06±12.14	0.268	56.62±13.75	56.30±12.01	0.890
**CTV-P**	67.21±10.86	64.08±17.02	0.306	79.62±16.52	67.48±14.04	0.004
**CTV-D**	77.47±11.81	65.63±13.08	<0.001	88.46±17.80	70.46±12.12	<0.001
**(CTV-A)-(CTV-N)**	13.5(9,18.5)	17(10,27)	0.055^b^	21(10.5,30.5)	19(9,27)	0.735^b^
**(CTV-P)-(CTV-N)**	31(24.75,37.25)	29(22,39)	0.297^b^	44(31.5,54.5)	30(22,38)	0.007^b^
**(CTV-D)-(CTV-N)**	42.15±9.65	32.72±12.60	<0.001	51(38.5,68.5)	33(26,39)	<0.001^b^
**(CTV-P)-(CTV-A)**	18(11.5,22.25)	10(4,18)	<0.001^b^	23(15,28.5)	9(6,16)	<0.001^b^
**(CTV-D)-(CTV-P)**	10(6.75,12)	4(-3,7)	<0.001^b^	7(4.5,13)	4(-2,8)	0.012^b^

CTV-N, CTV-A, CTV-P, and CTV-D CT values during nonenhanced, arterial, portal, and delayed phases, respectively.

a Fisher’s exact tests were applied to categorical variables, and chi-square tests were applied to all other variables.

b Mann-Whitney U test was applied to continuous variables, and Student’s t-test was applied to all other variables.

In the quantitative analysis, the LD/SD ratio values of gastric schwannomas were close to those of GISTs, and there were no significant differences between the two groups in the training (P=0.276) and validation (P=0.600) set cases. The values of CTV-D, CTV-D–CTV-N, CTV-P–CTV-A, and CTV-D–CTV-P in the gastric schwannoma group were significantly higher than those in the GIST group; there were significant differences between the two groups in the training (all P<0.001) and validation (P<0.001, <0.001, <0.001, = 0.012, respectively) sets. The CTV-N value (P=0.034) in the gastric schwannoma group was slightly higher than that in the GIST group in the training set. The values of CTV-P (P = 0.004) and CTV-P–CTV-N (P=0.007) in the gastric schwannoma group were slightly higher than those in the GIST group in the validation set. There were no significant differences in the values of CTV-A (P=0.268), CTV-P (P=0.306), CTV-A–CTV-N (P=0.055), or CTV-P–CTV-N (P=0.297) between the two groups in the training set, and there were no significant differences in the values of CTV-N (P=0.814), CTV-A (P=0.890), or CTV-A–CTV-N (P=0.735) between the two groups in the validation set.

### 3.4 Establishment of a nomogram model and validation of its predictive accuracy

**Figure 2:**
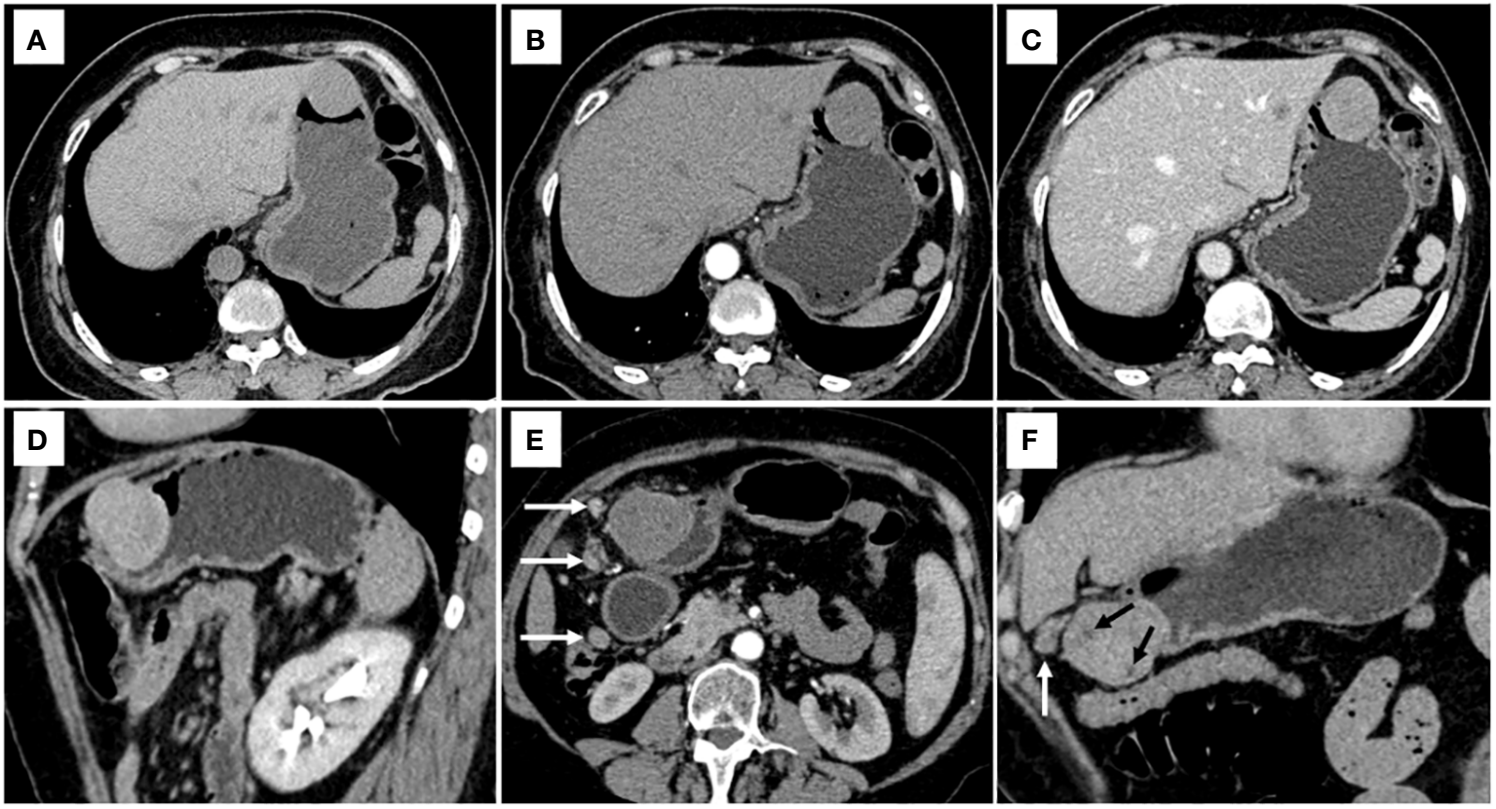
CT examination of patient 1, including axial unenhanced **(A)**, arterial phase image **(B)**, and portal phase image **(C)**, and oblique sagittal delayed phases image **(D)**, showed a mixed growth pattern lesion in the central of stomach without peritumoral lymph nodes and necrosis. The value of [(CTV-P) -(CTV-A)] and the value of [(CTV-D) -(CTV-P)] were 30 and 16, respectively. The nomogram accurately diagnosed gastric schwannoma with a predicted probability of 87%. CT examination of patient 2, including axial arterial phase image **(E)**, and coronal delayed phases image **(F)**, showed a mixed growth pattern lesion in the lower of stomach with the peritumoral lymph nodes (white arrow) and necrosis (black arrow). The value of [(CTV-P) -(CTV-A)] and the value of [(CTV-D) -(CTV-P)] were 15 and 16, respectively. The nomogram accurately diagnosed gastric schwannoma with a predicted probability of 92%. Finally, the tumors were confirmed as gastric schwannoma by histopathology.

**Figure 3:**
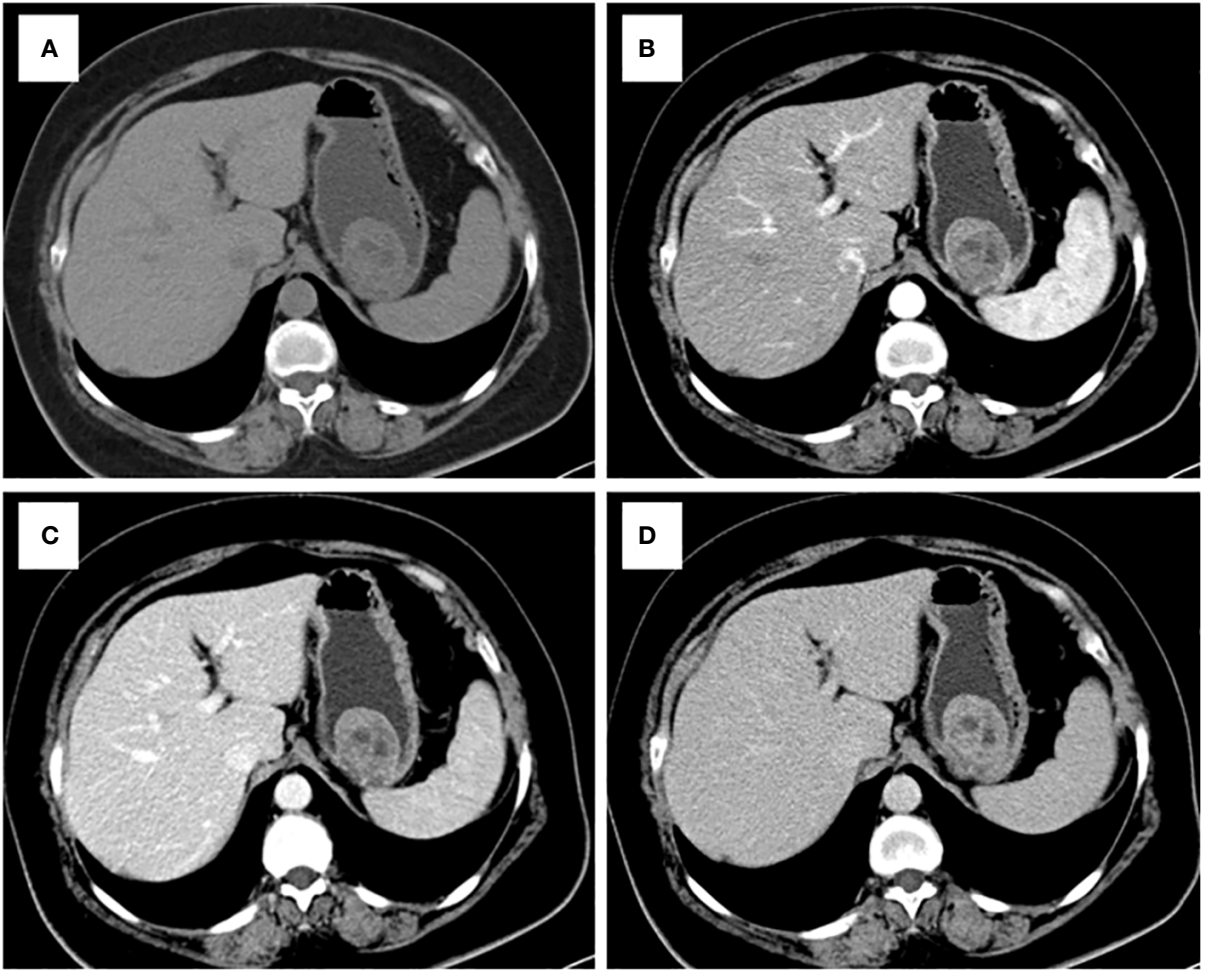
CT examination of patient 3, including axial unenhanced **(A)**, arterial phase image **(B)**, and portal phase image **(C)**, and delayed phases image **(D)**, showed an intraluminal growth pattern lesion in in the upper of stomach with necrosis. The value of [(CTV-P) -(CTV-A)] and the value of [(CTV-D) -(CTV-P)] were 9 and 12, respectively. The nomogram accurately diagnosed gastric schwannoma with a predicted probability of 4%. Finally, the tumors were confirmed as GIST by histopathology.

**Figure 4:**
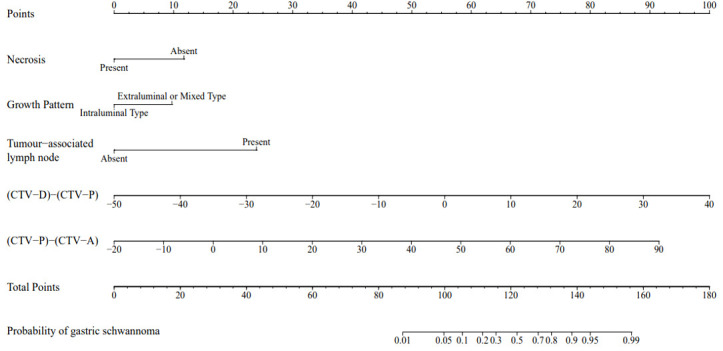
A nomogram was developed in the training set incorporating necrosis, growth pattern, tumor-associated lymph node, the value of [(CTV-D)-(CTV-P)], the value of [(CTV-P)-(CTV-A)].

**Figure 5:**
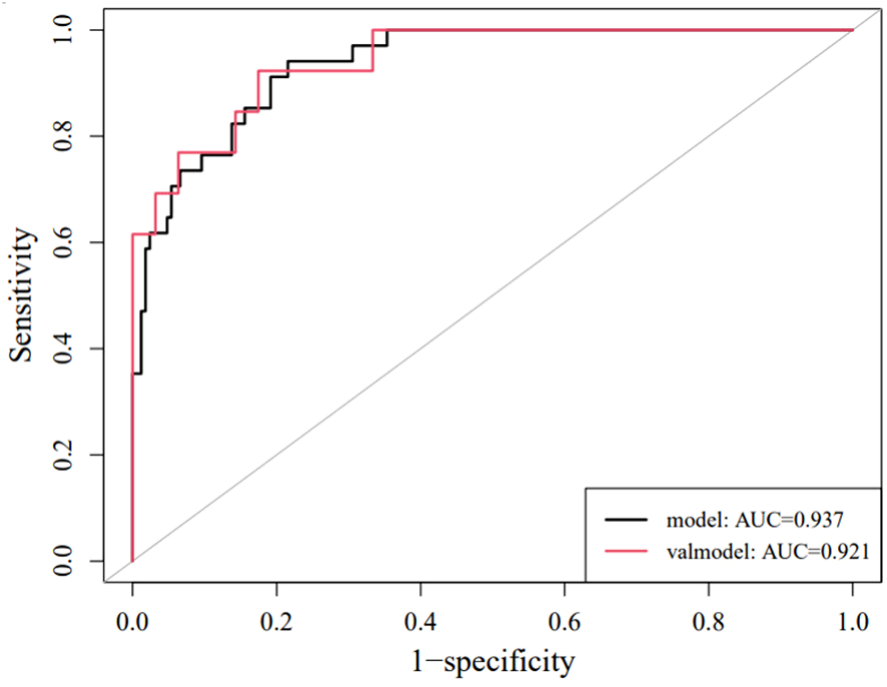
ROC curves of the nomogram in the training set and validation set.

**Figure 6:**
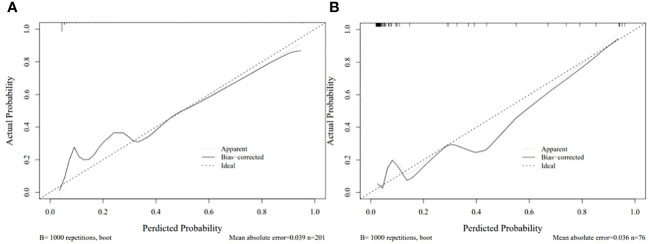
Calibration curve for the nomogram in the training set **(A)** and validation set **(B)**. .

Each statistically significant variable in the univariate analysis was subjected to collinearity and correlation assessments. Because of the enhancement pattern and necrosis, the values of CTV-D–CTV-N and CTV-D exhibited multicollinearity and obvious correlation; the enhancement pattern and the value of CTV-D were removed. Logistic regression analysis showed that extraluminal or mixed growth pattern (odds ratio [OR] 3.626; 95% CI 1.105–11.900; P=0.034), absence of necrosis (OR 4.752; 95% CI 1.464–15.424; P=0.009), presence of tumor-associated lymph nodes (OR 23.978; 95% CI 6.499–88.466; P<0.001), and the values of CTV-P–CTV-A (OR 1.117; 95% CI 1.042–1.198; P=0.002) and CTV-D–CTV-P (OR 1.159; 95% CI 1.080–1.245; P<0.001) were independent predictive factors associated with gastric schwannoma (**Figures 2**, **3** and **Table 4**). A nomogram model was also established (**Figure 4**). The final nomogram model yielded AUCs of 0.937 (95% CI 0.900–0.973) and 0.921 (95% CI 0.830–1.000) in the training and validation sets, respectively. The sensitivity, specificity, and accuracy of the nomogram model in the training set were 94.1%, 78.4%, and 81.1%, respectively, whereas those in the validation set were 92.3%, 82.5%, and 84.2%, respectively (**Figure 5**). The results of the Hosmer-Lemeshow goodness-of-fit test in the training (χ^2 ^= 3.501; P=0.899) and validation sets (χ^2 =^ 8.178; P=0.416) indicated that the calibration of the nomogram model was appropriate (**Figure 6**).

**Table 4:** Logistic regression analysis of CT features for prediction of Gastric schwannoma.

Constants and Variables	*β value*	Odds Ratio (95 %CI)	*P value*
**Growth Pattern(Extraluminal or Mixed Type)**	1.288	3.626(1.105~11.900)	0.034
**Tumour-associated lymph node (present)**	3.177	23.978(6.499~88.466)	<0.001
**Necrosis (absent)**	1.558	4.752(1.464~15.424)	0.009
**(CTV-P)-(CTV-A)**	0.111	1.117(1.042~1.198)	0.002
**(CTV-D)-(CTV-P)**	0.148	1.159(1.080~1.245)	<0.001
**Constant**	-6.602		<0.001

## 4 Discussion

In the present study, a quantitative description of tumor imaging features was added using five CT imaging features, including extraluminal or mixed growth pattern, absence of necrosis, presence of tumor-associated lymph nodes, and the values of CTV-P–CTV-A and CTV-D–CTV-P, treated as independent predictive factors for the differential diagnosis of gastric schwannoma and GIST (diameters = 2–5 cm) based on logistic regression analysis. The CT-based nomogram derived from these factors had a higher diagnostic efficiency, sensitivity, and specificity in both the training and validation sets. This visualized differential diagnosis nomogram model helps improve the accuracy in predicting tumor properties and provides a favorable basis for clinicians to choose surgical plans.

There were five predictive factors for imaging features that differentiated gastric schwannomas from GISTs in the training set. Compared with previous studies (3, 13), the presence of tumor-associated lymph nodes was also observed in our study, with an OR 23.978, thus indicating that the presence of tumor-associated lymph nodes is the most significant characteristic distinguishing gastric schwannomas from GISTs. Some authors (5, 13, 25, 26) have stated that the peritumoral lymph nodes around gastric schwannomas are manifestations of inflammatory reactive hyperplasia, and we agree with this view. In the training set, the extraluminal or mixed growth pattern and absence of necrosis were the other two independent factors (OR=3.626 and 4.752, respectively). In our study, gastric schwannomas mostly occurred in the middle part of the stomach (21/34, 61.76%), typically associated with extraluminal or mixed type (27/34, 79.41%), whereas GIST mainly occurred in the upper and middle parts of the stomach (150/167, 89.82%), with intraluminal growth (91/167, 54.49%), which is consistent with previous reports (13, 16, 27). In this study, gastric schwannomas rarely exhibited intralesional necrosis, and necrosis and calcification were more common in the periphery of the tumor compared with GISTs. Some studies have found that gastric schwannomas grow slowly and that neovascularization provides an adequate blood supply for their growth, thus resulting in rare necrosis; however, GISTs may be potentially malignant, and insufficient internal blood supply can lead to ischemia and necrosis of tumor cells (3, 5), which is consistent with the results of this study.

The results of this study showed statistically significant differences in the values of CTV-D, CTV-P–CTV-A, and CTV-D–CTV-P between gastric schwannomas and GISTs in the training and validation sets. Owing to the different vascularity profiles of gastric schwannoma and GIST, the degree of enhancement of gastric schwannoma was lower than that of GIST in the arterial phase. Previous studies showed that GIST is typically a hypervascular lesion on contrast-enhanced CT (5, 28). It has been suggested that the enhancement of gastric schwannomas occurs over time, with peak enhancement occurring during the delayed phase, which may be related to the slender blood vessels supplying the lesion, thus leading to the slow infiltration of contrast agents from the blood vessels into the surrounding tissue gaps (18, 29). Conversely, as previously mentioned, the value of CTV-D may decline in GIST because of the fast washout of intratumoral contrast agents. These reasons could explain the findings of the current study that showed that the values of CTV-P–CTV-A and CTV-D–CTV-P in gastric schwannomas were significantly higher than those in GISTs.

Wang et al. (16) compared and analyzed several different methods to differentiate gastric schwannomas and GISTs based on machine learning and stated that the logistic regression model could be robust and accurate. In this study, an intuitive visual nomogram model was constructed using five imaging feature predictors screened by logistic regression, which was confirmed to have good consistency with clinical practice in both the training set (AUC = 0.937) and the external validation set (AUC = 0.921). In clinical applications, the selected imaging feature prediction factors are easy to collect and can conveniently and quickly improve the ability of physicians to distinguish gastric schwannomas from GISTs.

This study has the following limitations. First, in the logistic regression analysis, combined classifications may have influenced the results owing to the small number of tumors with specific sites and growth patterns. Second, this was a retrospective study conducted in two centers only; the sample size was small and inevitably led to certain selection biases. Owing to the small sample size, the results were not sufficiently robust. In future studies, we plan to evaluate the reliability of the current nomogram using data from multiple centers and a prospective design.

In conclusion, this study developed and validated a diagnostic nomogram model based on CT imaging features that allowed the development of an accurate and non-invasive evaluation method for differentiating gastric schwannomas and GISTs. As the basis of the non-invasive semiquantitative examination method, the nomogram model can supplement conventional examination methods and assist clinicians in decision-making.

## Data availability statement

The original contributions presented in the study are included in the article/supplementary material. Further inquiries can be directed to the corresponding authors.

## Ethics statement

The studies involving humans were approved by Affiliated Hospital of Jining Medical University. The studies were conducted in accordance with the local legislation and institutional requirements. The participants provided their written informed consent to participate in this study.

## Author contributions

LZ: Conceptualization, Data curation, Funding acquisition, Writing – original draft, Writing – review and editing. GC: Data curation, Resources, Validation, Writing – original draft. ZS: Data curation, Resources, Validation, Writing – original draft. JX: Data curation, Resources, Validation, Writing – original draft. HY: Writing – original draft, Conceptualization, Formal Analysis, Supervision. ZW: Writing – original draft, Data curation, Resources, Validation. SM: Data curation, Resources, Validation, Writing – original draft. YC: Funding acquisition, Methodology, Resources, Software, Supervision, Writing – review and editing.
